# Supplementary material for: A retrospective view of the relationship of soluble Fas with anemia and outcomes in chronic kidney disease
Source: PLoS One. 2023 Jun 30;18(6):e0286854. doi: 10.1371/journal.pone.0286854 (PMC10313056; doi:10.1371/journal.pone.0286854)
Supplement: Attachment — Submitted filename: Comments on PLOS ONE article.docx [file pone.0286854.s001.docx]

PONE-D-23-00605 entitled: “A retrospective view of the relationship of soluble Fas with anemia and outcomes in chronic kidney disease”.

**Comments**

1- There are few grammar mistakes, confused sentences, and typos throughout the manuscript. It should be revised carefully.
2- The introduction should be one way or another to be extended to include dissertation about erythropoietin (EPO).
3- References should be updated in both the introduction and the discussion (lack of 2021 &2022 references).
4- Results are well organized, but correlations were not discussed in the discussion part of the manuscript.
5- The discussion should be extended; more other previous studies in the same area of the present study should be used to show that the present results match or do not with that of other previous studies.
6- The conclusion was limited only to diabetes mellitus, age, and higher serum sFas levels although the study includes several other variables such as Hb, serum albumin IL-6, IFN-γ, Serum EPO and iPTH which were not discussed well.
7- There are some weak points in the present study, which are the sample sizes of the patients, and the healthy volunteers who participated in the study were small as proven in the article by the authors. Also, the researchers did not carry out any intervention in
   the group of patients and they focused on patients with NDDCKD and serum levels of inflammatory markers (which were not referred to in the discussion) and sFas that are associated with anemia and hyporesponsiveness to EPO, with most of the patients already reported.
